# Chlorinated herbicides in fish, birds and mammals in the Baltic Sea

**DOI:** 10.1007/s11270-015-2536-x

**Published:** 2015-07-30

**Authors:** Andrzej R. Reindl, Lucyna Falkowska, Agnieszka Grajewska

**Affiliations:** Department of Marine Chemistry and Environmental Protection, Faculty of Oceanography and Geography, University of Gdansk, Pilsudskiego 46, 81-387 Gdynia, Poland

**Keywords:** S-triazine derivatives, EU priority substances, Fish-eating birds, Baltic mammals, Baltic Sea

## Abstract

The aim of the present work was to determine the concentration levels, as well as accumulation and magnification coefficients, of triazine derivatives in herring gulls and Baltic grey seals 11 years after a ban on their use in the EU and eight after their exclusion in Poland. Dead birds were collected in the coastal zone of the Gulf of Gdansk in the years 2010–2012. The grey seals, on the other hand, were from before 2007, when s-triazine derivatives were still in use. Triazine herbicides (atrazine, simazine, propazine, terbutrine, prometrone, prometrine and ametrine) were found in the muscles and livers of birds and mammals and also in fish. The obtained results indicated the presence of all the assayed triazines in whole Baltic herring and their livers, while fish muscles were found to be free of prometrone and ametrine. In the muscles and liver of the grey seal, no ametrine, propazine or terbutrine were found, while prometrine was found in the liver of only one specimen. Research showed that simazine did not accumulate and magnify in marine birds and mammals. Atrazine became accumulated in the liver of birds and mammals while magnification was determined in their muscles. The accumulation of ametrine was found in the muscles of seals.

## Introduction

The environmental pollution of the Baltic Sea with organic compounds is a problem frequently pointed out in scientific papers (Falkowska et al. 2013; Nödler et al. 2013; Reindl et al. 2013a, b; Usydus et al. 2009) as well as in HELCOM (2011, 2010) and EEA reports (2010). This group of harmful substances includes organic chlorine compounds such as pesticides, fungicides and herbicides, which are produced and used on land, then transported via rivers and the atmosphere to the marine environment. Symmetrical triazines (s-triazines) are organochlorine plant protection agents, which are effective in eliminating many species of weed, but at the same time, selective towards the crop. Triazine herbicides block the flow of electrons in photosystem II, which disrupts the process, resulting in the killing of the weed. These compounds undergo metabolic transformations in plants through oxidation or de-alkylation (Strong et al. 2002). A significant reason for the introduction and subsequent widespread use of triazines in agriculture was their affordability. After over 50 years of continual usage, often in excessive doses, negative effects were observed in the environment (Gołębiewska and Snopczyński 2007). Owing to the high solubility of herbicides, their usage in agrotechnical procedures resulted in their penetration into ground water (Sadowski 1996) and its contamination (Pollehne et al. 1999). S-triazine derivatives were found in atmospheric precipitation (Polkowska et al. 2000) and their significance in atmospheric deposition was confirmed by Bester et al. (1995).

On the basis of environmental monitoring, the EU Plant-Scientific Committee decided that triazine derivatives containing atrazine and its decomposition products exceeding the limits (0.1 μg dm^−1^) set out in Annex I to Council Directive 91/414/EEC, result in excessive pollution of ground water. Consequently, on 10 March 2004, a decision was made by the European Committee (EC 2004) to exclude atrazine from Annex I to Council Directive 91/414/EEC, which contains a list of substances that are acceptable for use in plant protection agents, and to withdraw permits for agents containing this substance starting from 10 September 2004. Owing to the widespread use of atrazine and its wide spectrum of influence, in order to avoid gaps in plant protection schemes, the Committee enabled some member states (including Poland) to conditionally withhold the permit for agents containing atrazine until 30 June 2007.

Despite the ban on the usage of s-triazine derivatives in agrotechnical procedures in the EU, the most recent studies have proven their continued presence in European seas (Nödler et al. 2013). Previous studies had investigated the effect of triazine herbicides on the lowest level of the marine trophic chain (Liu et al. 2009; He et al. 2012). Experimental studies showed that environmental exposition to s-triazine derivatives dissolved in water had a moderately toxic influence on fish and their larvae (Arufe et al. 2004; Velisek et al. 2010), while higher than environmental doses of these herbicides resulted in pathological changes in the internal organs of fish (Wang et al. 2011). S-triazines disrupt neurotransmissive flows (Filipov et al. 2007). Moore et al. (2007) found disruptions in the migratory activity of the Baltic salmon (*Salmo salar*) when exposed to atrazine. The endocrinological effect of s-triazine derivatives in the common quail (*Coturnix coturnix*) was confirmed by de la Casa-Resino et al. (2012). A few studies have shown that such chemicals can cause not only direct intoxication and obvious effects, such as the death of marine biota, but also more subtle negative effects such as impairment of the reproductive, hormone and immune systems.

Laboratory experiments and environmental analysis have shown that even small amounts of atrazine (0.1 ppb) can have adverse effects on certain species of amphibians, leading to hermaphroditism and delayed gonadal development (Hayes et al. 2003). Available publications on the influence of herbicides on marine organisms indicated “poor knowledge” and there is a general lack of such studies in the Baltic. The concentration levels of triazine herbicides in marine birds and mammals and the problems related to their accumulation are not present in publications; hence, the focus of the present analyses on organisms on the highest level of the trophic chain in the Baltic. These include tests on herring gulls (*Larus argentatus*)—omnivorous migratory birds found in and around the Gulf of Gdansk, a mammal—the grey seal (*Halichoerus grypus*) from the Baltic and specimens kept at the Marine Station of Gdansk University as part of a reintroduction programme. As piscivorous animals in zoos and reintroduction centres are fed Baltic herring (*Clupea harengus*), the present studies encompassed these fish as well. Moreover, the Baltic herring is also a very popular fish in terms of human consumption in Poland.

The chemical analyses conducted in this study included the assay of atrazine (1-chloro-4-ethylamino-6-isopropylamino-1,3,5-triazine); simazine (2-chloro-4,6-bis(ethylamino)-1,3,5-triazine); propazine (2,4-Bis(isopropylamino)-6-chloro-1,3,5-triazine); terbutrine (1,3,5-triazine-2,4-diamine); and prometrone (2,4-Bis-(isopropylamino)-6-methoxy-1,3,5-triazine); prometrine (2,4-Bis-(isopropylamino)-6-methylthio-1,3,5-triazine); and ametrine (2-ethylamino-4-isopropylamino-6-methylthio-1,3,5-triazine) in the livers and muscles of the analysed biota.

## Materials and methods

Studies were carried out on whole Baltic herring (*C. harengus*) caught in 2012 in the Gulf of Gdansk (*n* = 9) and also in the muscles (*n* = 7) and livers (*n* = 7) of these fish. Studies also included the muscles (*n* = 19) and livers (*n* = 19) of herring gulls (*L, argentatus*). The dead specimens were found in the years 2010–2012 close to the breeding colonies in Wladyslawowo (*φ* = 54° 47′ and *λ* = 18° 25′) and the Mewia Lacha Nature Reserve, situated close to the outlet of the River Vistula into the Gulf of Gdansk (*φ* = 54° 21′ and *λ* = 18° 57′). The muscles (*n* = 8) and livers (*n* = 8) of grey Baltic seals (*H. grypus*) were also subject to chemical analysis, the dead specimens originating from the seal sanctuary at the Marine Station belonging to Gdansk University’s Institute of Oceanography (2001, 2005), and from the beaches of the Gulf of Gdansk before 2007.

### Reagents and standards

To determine triazine derivatives in biological material, organochlorine herbicide standards supplied by Supelco (high-performance liquid chromatography (HPLC) area ≥99.5 %) were used. Acetonitrile, methanol, ultra pure water and ammonium acetate (HPLC area ≥ 99 %) used for extraction and chromatographic analysis, were supplied by MERCK. In the procedure, the following were used: solid-phase extraction (SPE) columns with Thermo Scientific HyperSep type octadecyl-C18 cartridges and pure nitrogen: 99.994 % from Linde.

### Preparation and determination

Herbicide extraction was carried out from lyophilised material with a methanol/acetonitrile mixture (3:2) using the sonification method in three 45-min cycles. The extracts were purified using the SPE method on octadecyl-C18 cartridges (Muñoz and Rosés, 2000; McLaughlin et al. 2008; Fenoll et al. 2011).

The assay of s-triazine derivatives was carried out using an Agilent 1200 high-performance liquid chromatography (HPLC) system, equipped with DAD and RID detectors. The division of analytes was conducted on a Fusion 5-μm Phenomenex column. The mobile phase was a mixture of acetonitrile/0.1 M ammonium acetate in the proportions 33:67 in isocratic conditions (Tekel and Kovačičová 1993; Pinto and Jardim 2000; Özhan et al. 2005).

### Quality control

The limit of detection for herbicides was 3–12 ng g^−1^ dry weight. The herbicides were recovered by adding the known standard concentrations for the analysed substances to the studied biological material before its extraction. The analytical method used made it possible to recover between 79 and 87 % of the substance. In the analytical procedure, triple quantification of the blank sample was used.

Calculations:

- the bioaccumulation factor (BAF):
  1 $$ \mathrm{B}\mathrm{A}\mathrm{F}=C/{C}_{\mathrm{F}} $$
  where *C* is the concentration in the particular tissue or organs and *C*_F_ is the concentration in whole fish;
- the biomagnification factor (BMF):
  2 $$ \mathrm{B}\mathrm{M}\mathrm{F}=C1/C2 $$
  where *C*1 is the concentration in the tissue or organs of predators and *C*2 is the concentration in the analogous tissue or organs of herring

## Study results

**Fig. 1:**
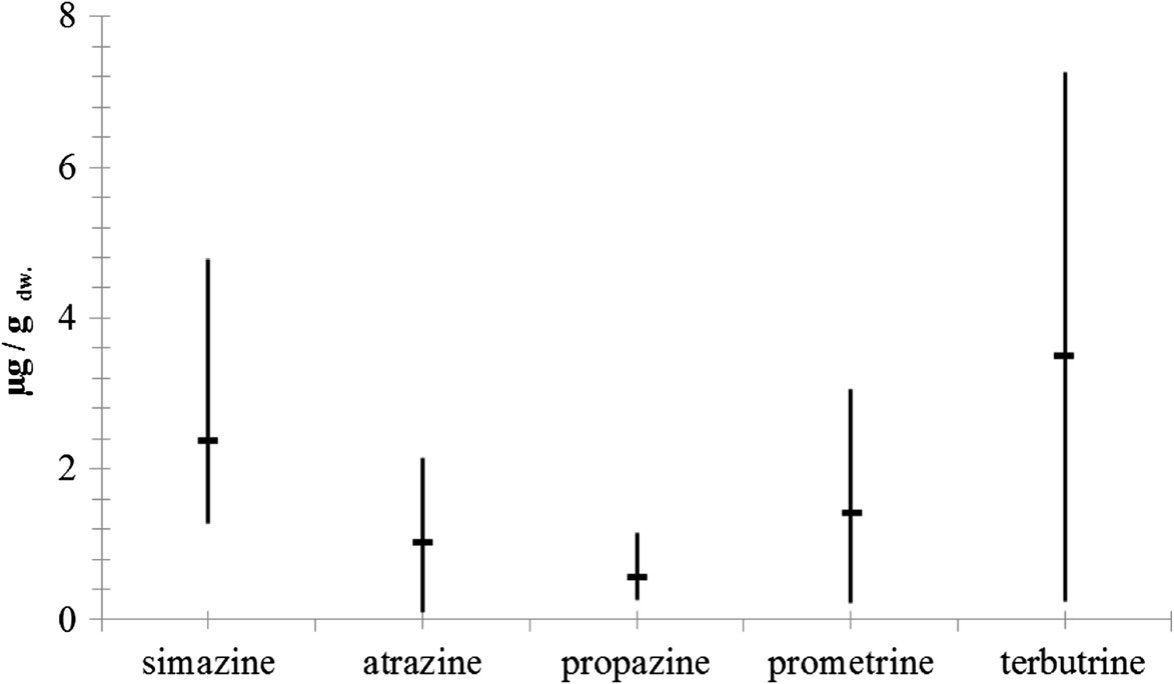
Average and range concentration of s-triazine derivatives in whole Baltic herring

Residue of s-triazine derivatives was found in whole Baltic herring (Fig. 1), one of the key food sources for marine birds and mammals in the Southern Baltic. All of the studied herbicides were found in the livers of these fish, while the muscles were found to be free of prometrone and ametrine. The presence of prometrone (0.17 μg g^−1^ dw) and ametrine (0.21 μg g^−1^ dw) was noted in only one sample containing a whole fish (Table 1).

**Table 1:** S-triazine derivative concentrations [μg g^−1^ dw] in the liver of analysed biota ± standard deviation, and liver versus muscle partition factor (L/M) in Baltic biota

Compounds	*Clupea harengus*		*Larus argentatus*		*Halichoerus grypus*
	Liver	L/M	Liver	L/M	Liver
Simazine	2.2 ± 0.8	1.8	0.5 ± 0.4	2.2	0.8 ± 0.6
Atrazine	1.7 ± 0.4	5.3	1.3 ± 1.0	1.9	1.3 ± 0.8
Prometrone	0.2 ± 0.04	–	3.2 ± 2.9	1.5	0.3 ± 0.1
Ametrine	0.1 ± 0.02	–	1.1 ± 0.9	1.7	<0.1
Propazine	0.4 ± 0.3	1.6	0.7 ± 0.9	1.1	<0.1
Prometrine	0.6 ± 0.4	0.9	1.5 ± 1.3	0.7	0.8
Terbutrine	1.8 ± 0.6	1.2	4.9 ± 5.7	1.4	<0.3

– lack of data for calculation, < data below detection limit

All of the herbicides were found in the muscles and livers of herring. In the muscles and liver of the grey seal, however, no ametrine, propazine or terbutrine was determined, while prometrine was discovered in the liver of only one specimen (Table 1).

The obtained values for the bioaccumulation factor (BAF) and biomagnification factor (BMF) show that simazine does not undergo accumulation and magnification in marine birds and mammals (Table 2). The highest accumulation factors out of all the s-triazine derivatives were calculated for prometrone in the muscles and livers of birds. In mammals, it was found that ametrine accumulated in the muscles of seals, while prometrine did not accumulate in the livers of mammals, but still underwent magnification.

**Table 2:** Accumulation (BAF) and magnification factors (BMF) in the muscles and liver of *L. argentatus* and *H. grypus* from the Southern Baltic collected before 2007

Compounds	Bioaccumulation factor (BAF)				Biomagnification factor (BMF)		
	*Larus argentatus*		*Halichoerus grypus*		*Larus argentatus*		*Halichoerus grypus*
	Muscle	Liver	Muscle	Liver	Muscle	Liver	Muscle
Simazine	0.1	0.2	0.2	0.3	0.2	0.2	0.4
Atrazine	0.7	1.3	0.7	1.2	2.1	0.8	2.2
Prometrone	12.3	18.6	1.3	1.4	–	17.6	–
Ametrine	3.2	5.4	1.3	–	–	12.6	–
Propazine	1.1	1.2	–	–	2.4	1.6	–
Prometrine	1.5	1.0	–	0.5	3.0	2.3	–
Terbutrine	1.0	1.4	–	–	2.3	2.8	–

## Discussion

For many years, herbicides have been widely used on all continents (Pempkowiak et al. 2000; Wang et al. 2011; Velisek et al. 2012). However, in the EU member states, owing to their toxic influence and the number of harmful effects on plant and animal organisms, atrazine and simazine (both belonging to the symmetrical triazine group) were banned from use starting from 2004 (EC 2004). Years before, in 1991, the same ban had been introduced in Germany and Italy. Atrazine has been classified as an endocrine compound and, alongside simazine, is treated as a priority substance by the EU water policy (HELCOM 2010). The latest research has shown that atrazine concentrations have dropped 20-fold since the introduction of the ban, despite the compound’s constant presence in the coastal waters of Northern and Southern Europe (Nödler et al. 2013). At the end of the twentieth century, the concentrations of atrazine, simazine and terbutrine found in the Western Baltic ranged from 1 to 13 ng dm^−3^ (Bester and Hühnerfuss 1993). Similar concentration levels were determined for the same s-triazine derivatives (4–19 ng dm^−3^) in the open part of the Baltic Sea and in the Gulf of Pomerania and the Gulf of Gdansk (Pempkowiak et al. 2000). Recent research, conducted nearly 20 years after the xenobiotics were withdrawn from use in Germany, has confirmed the presence of atrazine in the water of the western coast of the Baltic (Nödler et al. 2013). Researchers highlight the fact that the highest concentrations of these compounds are observed in the coastal zone of the sea when there is increased run-off water from land following intense precipitation and floods, and after agrotechnical procedures performed in spring and autumn. The good solubility of s-triazine enables the compounds to penetrate into surface and ground water faster than hydrophobic pesticides. Their subsequent transportation into the sea via rivers takes place together with other organic and inorganic pollutants in their dissolved or suspended forms (Bester et al. 1995). The contamination of rivers translates to a potential reduction of survival rates for organisms on all trophic levels. Ranke-Rybicka et al. (1995) showed that surfactants (anion detergents not exceeding the accepted concentrations for surface water) introduced with herbicides via the Vistula, by reducing surface pressure, make it easier for triazine herbicides to penetrate through semi-permeable cell membranes, and thus increase their harmful impact on water organisms (plankton, larvae, fish).

Triazine herbicides have the capacity to form complexes with trace metals, including permanent bonds, whose formation is dependent on environmental acidity (Grzesiak et al. 2007). The presence of trace metals in the water of the Baltic has been frequently discussed in literature (Szefer 2002), while the presence of trace metals in the discussed species of fish (Polak-Juszczak 2009), gulls (Szumiło et al. 2013) and seals (Kakuschke and Prange 2007) has also been confirmed. Despite the lack of experimental research into the influence of trace metals and triazine herbicides on organisms in the marine trophic chain, one cannot rule out synergic toxic action that exacerbates the adverse effect in the environment.

Fish are the most frequently considered organisms when assessing the potential risk related to the contamination of habitats (Usydus et al. 2009; Polak-Juszczak 2009; Szlinder-Richert et al. 2011; Reindl et al. 2013a). This is due to their direct exposure to chemicals which enter seawater as a result of agricultural activity, or which are directly introduced via the trophic chain. Birds, mammals and humans are all end consumers of fish, which is why the presence of atrazine and other triazine derivatives in the Baltic herring (in quite large amounts in relation to the other analysed herbicides—Table 1), suggests that either the EU ban is not strictly observed or that there are other routes of transportation introducing them from other sources. Seeing as herbicides have a limited lifetime in water, lasting up to a few months (Brzozowska 2004), their presence in the sea and in related organisms should be a minimal of 8 years after the introduction of the ban in Poland, and 11 years after its implementation in the EU. Other researchers have also shown that, despite its EU-wide ban in 2004, the triazine herbicide atrazine and its transformation products are frequently detected in the aqueous environment (in coastal waters of the Northern Aegean Sea, the Dardanelles, the Baltic Sea (Germany), the Northern Adriatic Sea (Italy), and the coastline close to Barcelona (Spain)). However, concentrations and detection frequencies in the coastal waters of the Baltic Sea (Germany) were low, indicating the success of the prohibition (Nödler et al. 2013). One cannot rule out the possibility of atrazine and simazine being transported by the wind and deposited onto the surface of the sea. The presence of pesticides in rainfall in Europe was reviewed by Dubus et al. (2000), who, in seeking to identify the origins of pesticides in air pollution, concentrated on their agricultural usage. They also pointed out, though, that there is evidence of non-agricultural pesticide use making a significant contribution to atmospheric input, transport and subsequent deposition. A likely explanation of triazine presence in the sea may be its introduction via rivers, especially during floods. During anomalous meteorological-hydrological phenomena, pollutants which have been deposited on land for decades become remobilised and transported into the marine environment. What makes the process even more disadvantageous is that a relatively large load of labile pollutants, often toxic, reaches the coastal zone of the sea, posing a potential threat to organisms in that region. The size of the pollutant load, particularly mercury, transported with the flood wave on the Vistula into the Southern Baltic in 2010, is described by Saniewska et al. (2014). The consequences of the introduction into the sea of s-triazine derivatives used on land in commercial weed killers are reflected by the statistically significant (*p* < 0.05) Spearman’s correlation coefficients calculated. In seals, a relation was discovered between atrazine and simazine in muscles (*r* = 0.92), between atrazine and prometrone in the liver (*r* = 0.90), and between atrazine in liver and simazine in muscles (*r* = 0.94). In the herring gull, there was a very strong correlation in muscles between atrazine and prometrone (*r* = 0.94) and in liver between atrazine and propazine (*r* = 0.97), ametrine and propazine (*r* = 0.89) and atrazine and prometrine (*r* = 0.66). The correlations in fish, however, were not statistically significant. It is worth emphasising that the herbicides used in Poland were mixtures of many of the studied triazines. The most commonly used combinations were the following: atrazine and simazine; atrazine and terbutrine and prometrine, atrazine and terbutrine. The chemical properties of triazines introduced into the environment determined their inclusion in the trophic chain and accumulation on the particular levels.

**Fig. 2:**
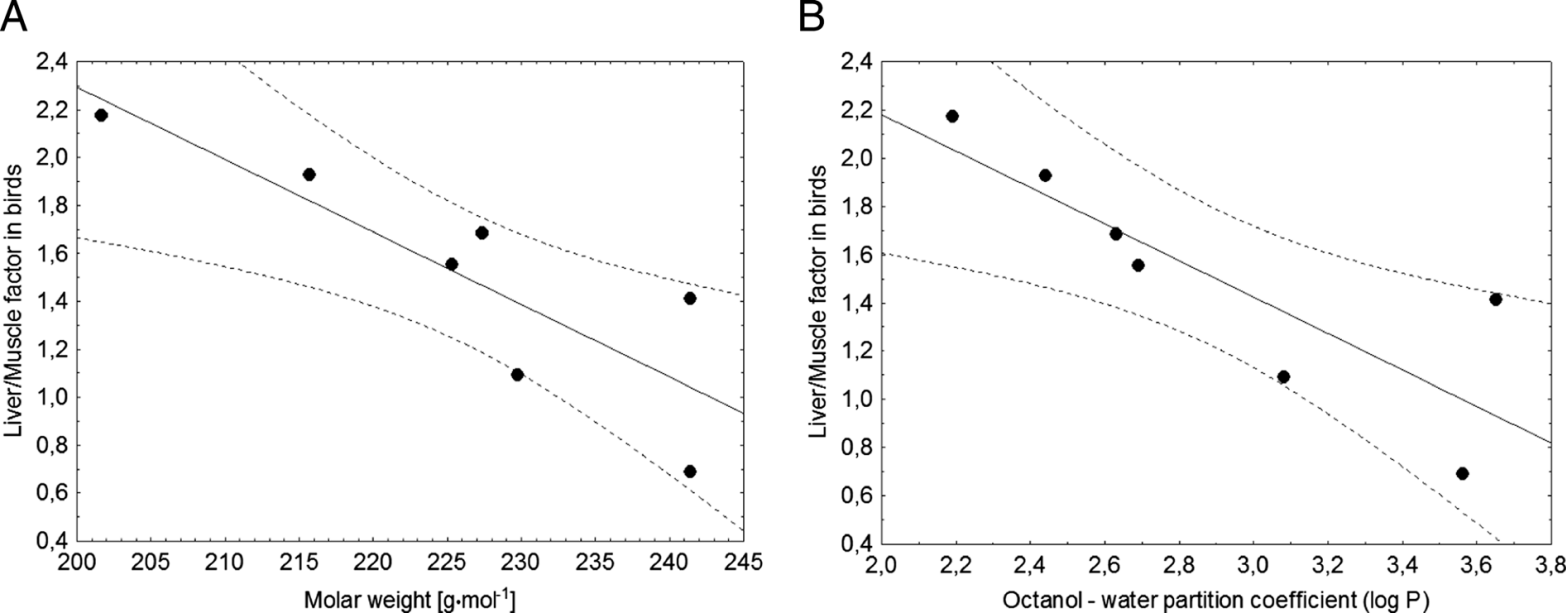
The dependency between the proportions of triazine concentrations in liver and muscles and molar weight (*y* = 8.3447–0.0303*x*; *r* = (−0.8499); *r* ^2^ = 0.7223; *p* = 0.0154) (**a**) and the octanol/water partition coefficient (*y* = 3.693–0.7562*x*; *r* = (−0.8418); *r* ^2^ = 0.7086; *p* = 0.0175) (**b**)

Apart from being purely cognitive, the studies into the bioaccumulation of environmental pollutants in the marine trophic chain aim to assess the degree to which top predators (including humans) are exposed. Many aquatic organisms exhibit the tendency to bioaccumulate compounds of a lipophilic nature, among these chloro-organic pesticides and herbicides (Reindl et al. 2013a, b; Falkowska et al. 2013). The accumulation and magnification factors for s-triazine derivatives were low compared to lipophilic organic pollutants of the marine environment. Despite the fact that the BAF factors indicated the potential for bioaccumulation in the trophic chain, the accumulation was not considerable (US EPA 1991). It appears that this was closely linked to the low division coefficient between octanol/water (atrazine *K*_O/W_ = 2.5 at 20 °C). It is worth emphasising that the livers of fish, gulls and seals were characterised by higher herbicide concentrations than the muscles, which could indicate that the studied organisms obtained the triazine derivatives with food, but that not all herbicides became accumulated. It was mainly prometrone and ametrine that were found to have accumulated in the liver and muscles of the herring gulls. What is more, biomagnification of both the herbicides was only observed in the livers of gulls. The sequestration of s-triazine derivatives in bird livers is indicated by the liver/muscle ratios, which were always higher than 1, with the exception of prometrine. Selective preservation in liver was determined by the molar weight and the octanol/water partition coefficient (Fig. 2)

It was thus easier for the liver to metabolise triazines with higher molar mass and greater affinity to lipids. In grey seals and fish, the relations of liver/muscle ratios of s-triazine derivatives to molar weight and octanol/water partition coefficients were not statistically significant. However, the bioaccumulation (BAF) and biomagnification factors (BMF) were close to 1 or else, impossible to quantify owing to concentrations < LOQ. Such a discrepancy probably resulted from the varying diet of gulls, consisting mainly of landfill waste supplemented with fish and fish guts discarded from fishing boats, and its less-efficient removal. Seals, on the other hand, only obtain food from the sea and this probably contains lower amounts of herbicides than communal waste on land.

## Conclusions

The obtained results indicated constant presence of all the assayed triazines (atrazine, simazine, propazine, terbutrine, prometrone, prometrine and ametrine) in whole Baltic herring and their livers. The muscles of herring, however, were free from prometrone and ametrine. In the muscles and liver of the grey seal, which feeds on herring, no ametrine, propazine or terbutrine were found, while prometrine was detected in the liver of just one specimen. Studies showed that simazine did not undergo accumulation and magnification in herring gulls or seals. Atrazine accumulated in the livers of birds and mammals, while its magnification was determined in their muscles. The accumulation of ametrine was observed in seal muscles.

The triazine herbicides selected for the study are “obsolete” plant protection agents, which have not been used in Polish agriculture for 8 years, but whose remnants are still present in the marine environment. An explanation of this fact may be atmospheric deposition and inflow via rivers, which wash out triazine remnants from the soil. This process is not continuous and its intensification can occur not only during springtime thaw but also during floods. Floods always affect a wide area on land and can result in a large load of pollutants being introduced with the flood wave into the coastal zone of the sea.
